# Torrefaction of Sericulture Agro-Industrial Waste for Sustainable Waste-to-Resource Solutions

**DOI:** 10.1021/acsomega.5c02508

**Published:** 2025-06-13

**Authors:** Edgar A. Silveira, Romulo C. Dutra, Júlia R. Vargas, Jefferson S. Oliveira, Paulo A. Z. Suarez, Grace F. Ghesti

**Affiliations:** † 28127 University of Brasilia, Mechanical Sciences Graduate Program, Laboratory of Energy and Environment, Brasilia 70910-900, DF, Brazil; ‡ 28127 University of Brasilia, Chemistry Institute, Laboratory of Brewing Bioprocesses and Catalysis to Renewable Energy, Brasilia 70910-900, DF, Brazil; § 28127 University of Brasilia, Chemistry Institute, Laboratory of Materials and Fuels, Brasilia 70910-900, DF, Brazil; ∥ Instituto Federal de Educação Ciência e Tecnologia de Brasília, Campus Gama, Brasilia 72429-005, DF, Brazil

## Abstract

The valorization of agro-industrial residues through torrefaction provides a sustainable pathway for biofuel production while mitigating environmental impacts associated with waste disposal. This study investigates the torrefaction of sericulture waste, specifically integral silkworm pupae (ISP) and lipid-extracted silkworm pupae (ESP), to assess the effect of lipid removal on biocoal properties. Initially, the ISP and ESP were subjected to torrefaction under various conditions (180–300 °C, 20–60 min) to compare their thermal behavior and fuel characteristics. Proximate, ultimate, and calorific analyses were conducted to evaluate changes in composition, fixed carbon content, and heating value. Results indicated that the ESP exhibited superior carbon retention and energy densification, supporting its selection for process optimization. A response surface methodology using a categorical central composite design (α = 1.41421) was then applied to optimize torrefaction conditions for ESP, considering temperature and residence time as key variables. Statistically significant models (*p* < 0.001) were obtained for solid yield and higher heating value (HHV), presenting reduced cubic and two-factor interaction models. The optimal torrefaction conditions (255 °C, 60 min) resulted in a biocoal with a solid yield of 83.13%, HHV of 22.12 MJ·kg^–1^, and an energy densification of 1.27. From an environmental perspective, torrefaction contributes to waste reduction and promotes biocoal quality. Economically, the process adds value to sericulture residues by converting them into high-energy biofuels, supporting decentralized energy solutions. The results support ESP residues as biofuel, highlighting the need for further research on the biorefining of extracted lipids, combustion performance, upscaling feasibility, and life cycle assessment to evaluate sustainability.

## Graphical abstract

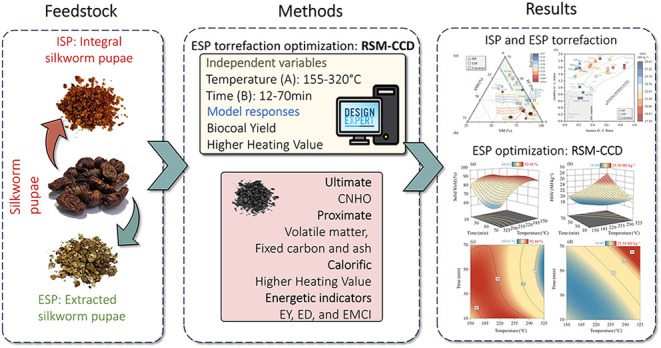

## 1 Introduction

The transition to sustainable energy demands diversifying biomass sources beyond conventional wood-based materials incorporating agro-industrial residues to enhance resource efficiency and energy generation.^1^ Agro-industrial residues are usually disposed of without treatment, resulting in socio-environmental issues.^2^ To address issues caused by the improper disposal of these residues, proposals suggest their reuse in more sustainable production systems, enhancing industrial economic viability.^3^

Economically, insects and their derived products present significant potential in low-income nations. Insect biorefinery is emerging as a valuable tool in the circular bioeconomy.^4^ Secondary products such as biodiesel and biochar can also be produced, serving as an energy source and assisting in organic waste management.^4^

The agro-industries of sericulture focus on using silkworm cocoons to extract continuous protein filaments and subsequently process those into silk.^5^ Due to the ease of propagation and the high added value of silk in the textile industry, sericulture is an excellent alternative for extractivist cultivation. As a coproduct of silk production, the silkworm pupae removed from their cocoons have underexplored industrial applications.

Sericulture waste presents valuable, yet underexplored, opportunities for sustainable purposes. Manjunath et al.^6^ have reviewed possible pathways for valorizing these residues. Precocoon residues, such as larval excreta and leftover leaves, can be processed into organic compost and vermicompost, enriching soil fertility and serving as a sustainable agricultural input.^6^ Moreover, silkworm pupal waste contains high protein and lipid content, making it suitable for animal feed production and the extraction of bioactive compounds with pharmaceutical applications.^6^ Regarding bioenergy applications, mulberry silkworm defatted pupae, litter, and excreta have been proposed as high-quality feedstock for biogas production, benefiting from their optimal C/N ratio and biodegradability.^6^ Despite this bioenergy valorization pathway, no study has investigated the effects of thermochemical routes on silkworm pupal waste.

Dutra et al.^7^ have investigated the potential of silkworm pupae extracted oil as a sustainable lubricant, converting it into polyols for eco-friendly grease formulations through epoxidation and hydrolysis, reinforcing its value as a high-impact alternative within circular economy frameworks. Building on this foundation, this study explores a novel valorization pathway, emphasizing the pre-extraction of lipid oil for further industrial applications and the subsequent utilization of the postextraction solid residue. In this context, this work aims to expand the potential of the postextraction solid residue as biocoal, enhancing resource efficiency and sustainability.

Torrefaction has been recognized as a viable strategy with increasing technology readiness level (TRL) for mitigating the inherent challenges associated with biomass utilization,^8,9^ thus presenting a promising avenue for valorizing underutilized feedstocks.^10,11^ This process is typically conducted within the temperature range of 200–300 °C and under controlled conditions, such as an inert or oxygen-lean atmosphere.^12^ Torrefaction imparts desirable attributes, the foremost among them being an increased energy density, which can be strategically harnessed to enhance biomass resource management.^13^ One of the objectives of torrefaction is to modify biomass to enhance its properties, making it more comparable to coal as a solid fuel (biocoal).^14^

Different tools are applied to assess the torrefaction outcomes and optimize the process for its utilization. In optimizing thermochemical conversion processes, researchers employ various statistical techniques, including factorial design, Taguchi method, response surface methodology (RSM), and analysis of variance (ANOVA).^12^ Regarding RSM, the technique offers optimization analysis, providing insights into the influence of individual factors on the response and explaining the intricate relationships between them.^15^ Moreover, it delivers results visualization and a comprehensive understanding of the optimization process. In addition, RSM includes an ANOVA, a statistical tool for assessing the relative significance of the operational conditions or torrefaction impacting system performance and biocoal quality.^16^

Studies on nonlignocellulosic materials for biochar are not as abundant as those of lignocellulosic biomass.^17^ Biochar samples from nonlignocellulosic sources are often prepared by pyrolytic routes, tested on soil remediation and adsorption, and not as often as biofuel.^18^ Specific biochar materials prepared from nonlignocellulosic biomass sources, mainly chitinous (insects) and nitrogen-rich residues, are compiled in Table 1, along with their treatment conditions, applications, and optimization approaches. Although numerous studies have explored the pyrolysis and torrefaction of various nonlignocellulosic feedstocks,^17^ Table 1 highlights the limited attention given to chitinous and nitrogen-enriched biomasses in biochar and biocoal production. Furthermore, this reveals that no previous study has assessed the torrefaction of silkworm pupae by applying RSM to analyze, predict, and optimize torrefaction outcomes.

**Table 1:** Overview of Pyrolysis and Torrefaction Applied to Chitinous and Nitrogen-Rich Biomass Residues, Emphasizing Applications (Adsorption and Biofuel) and Process Optimization

waste	treatment conditions	assessment/optimization	refs
adsorption			
ahrimp (*Penaeus brasiliensis*) chitin waste	**pyrolysis** /	adsorption of methyl violet dye solution	^19^
	atmosphere: N_2_	SY–27.5%	
	sample: 50 g	SSA–275 m^2^·g^–1^	
	temperature: 800 °C	Pore vol. –0.178 cm^3^·g^–1^	
	heating rate: 10 °C·min^–1^	*q*_e_^a^_(700 °C)_ –95.74 mg·g^–1^/	
	time: 60 min	**none**	
silkworm (*Bombyx mori*) frass waste	**pyrolysis** /	adsorption of Pb (HNO_3_)_2_, Cu (NO_3_)_2_, and Cd (NO_3_)_2_ solutions	^20^
	atmosphere: N_2_	SSA –68.2 m^2^·g^–1^	
	temperature: 600 °C	pore vol. –0.083 cm^3^·g^–1^	
	heating rate: 5 °C·min^–1^	*q*_e(Pb)_ –9.33 mg·g^–1^	
	Time: 120 min	*q*_e(Cd)_ –30.14 mg·g^–1^	
		*q*_e(Cu)_ –90.02 mg·g^–1^/	
		**none**	
bean-worm (*Clanis bilineata*) larvae skin waste	**pyrolysis** /	adsorption of Pb solution	^21^
	atmosphere: N_2_	SY –25.9%	
	temperature: 300–700 °C	SSA –36.5 m^2^·g^–1^	
	heating rate: 5 °C·min^–1^	pore vol. –0.039 cm^3^·g^–1^	
	Time: 240 min	*q*_max_^b^_(700 °C)_ –77.52 mg·g^–1^/	
		**none**	
black soldier fly (*Hermetia illucens*) larvae and pupae waste	**pyrolysis** /	adsorption of Pb (HNO_3_)_2_, Ni (NO_3_)_2_, and Cd (NO_3_)_2_ solutions	^22^
	atmosphere: N_2_	SSA –4.66 m^2^·g^–1^	
	temperature: 500–700 °C	pore vol. –0.021 cm^3^·g^–1^	
	Time: 30 min	*q*_max(Pb)_ –10.05 mg·g^–1^	
		*q*_max(Ni)_ –7.38 mg·g^–1^	
		*q*_max(Cd)_ –9.19 mg·g^–1^/	
		**none**	
cricket (*Acheta domesticus*) waste	**pyrolysis** /	*Lepidium sativum ro*ot elongation and seed germination inhibition	^23^
	atmosphere: N_2_	SY –22.3%	
	temperature: 500–700 °C	SSA –0.04 m^2^·g^–1^/	
	time: 60 min	**none**	
biofuel			
sewage sludge	**pyrolysis** /	SY –51.88%	^24^
	atmosphere: N_2_		
	sample: 10 g	HHV –12.44 MJ·kg^–1^	
	temperature: 450 °C	EY –39.13%/	
	heating rate: 10 °C·min^–1^	**none**	
	time: 30–90 min		
silkworm pupae waste	**torrefaction** /	solid yield (SY), proximate (FC, VM, and ASH) and ultimate analysis (CNHO), higher heating values (HHV), energy yield (EY), energy-mass coefficient index (EMCI) /	study
	atmosphere: N_2_	**RSM-CCD**	
	sample: 3 g		
	temperature: 180–300 °C		
	heating rate: 10 °C·min^–1^		
	time: 20–60 min		
	influence of lipid extraction		

a *q*_e_ - Adsorption Capacity.

b *q*_max_ - Maximum Adsorption Capacity.

In this context, the originality of this work lies in (i) proposing and evaluating, for the first time, the feasibility of producing biocoal from agro-industrial waste in sericulture through torrefaction, thereby contributing to a deeper understanding of integrated systems within the broader biorefinery and waste-to-resource framework; (ii) assessing the influence of lipid extraction on the torrefaction outcomes of silkworm pupae, providing insights into the quality of the residue postextraction and its potential for further valorization to enhance overall economic viability; and (iii) optimizing the torrefaction process of lipid-extracted silkworm pupae residue using RSM, with a focus on maximizing solid yield and calorific value to improve biocoal quality.

## 2 Materials and Methods

### 2.1 Feedstock

Brazil is currently the fifth largest producer of silk, with most production concentrated in the state of Paraná.^25^ In 2020, Brazilian farmers produced 377 tons of silk.^26^ To produce 1 kg of silk, 6.3 kg of silkworm cocoons are required.^26^ The silkworm pupae (silkworm residue) were sourced from silk producers in São Paulo, Brazil. The pupae, used as animal feed protein supplements, were dehydrated before being shipped and stored at low temperatures. Figure 1a shows the framework of the proposed analysis.

**Figure 1:**
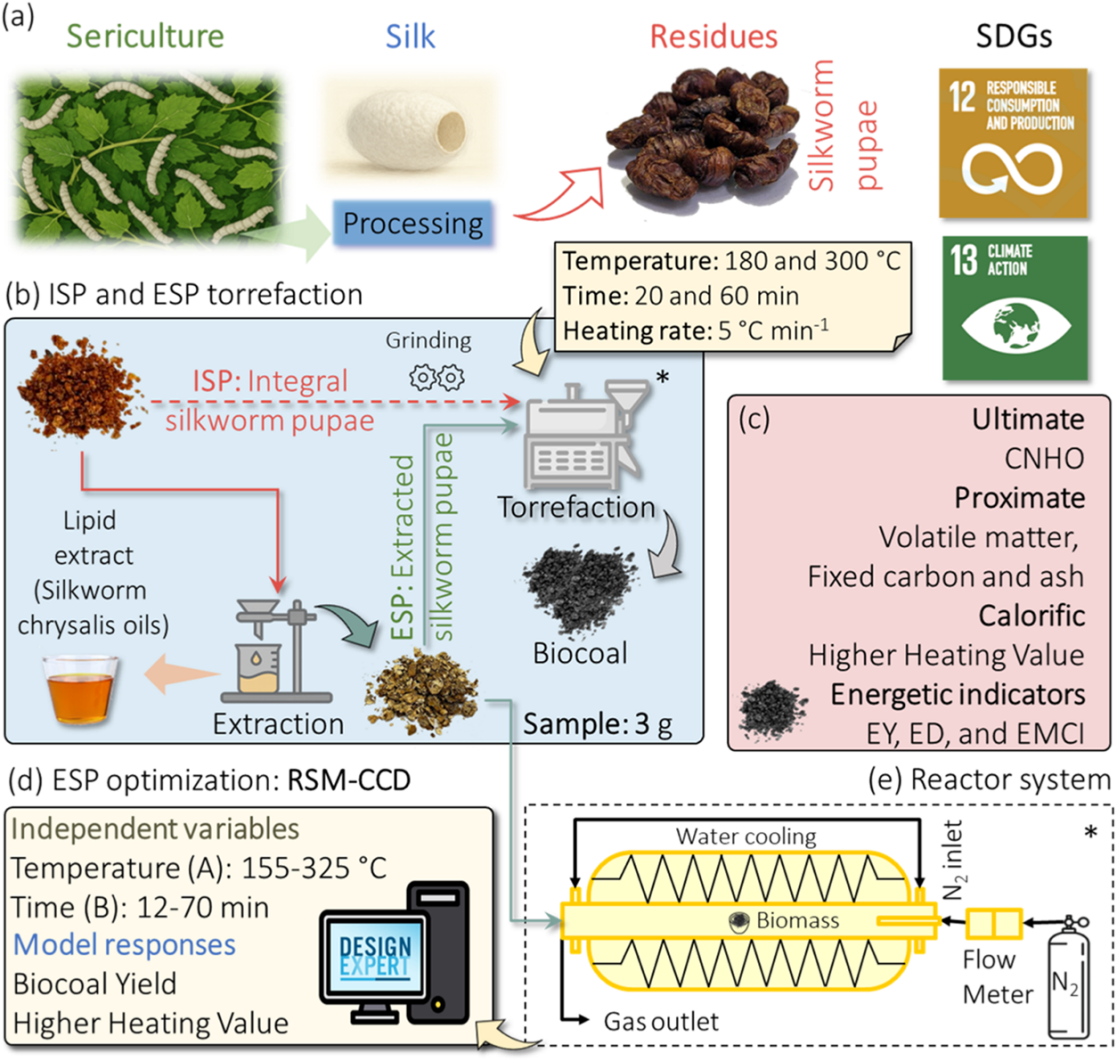
Research framework. (a) Sericulture and residue production. (b) Assessment of integral silkworm pupae (ISP) and post lipidic extraction (ESP) sampling and torrefaction. (c) Biocoal characterization. (d) Optimization (RSM-CCD) of torrefaction parameters using ESP feedstock and (e) torrefaction reactor system design and operation.

First, integral silkworm pupae (ISP) and the silkworm pupae post lipidic extraction (ESP) were assessed to evaluate the torrefaction influence of lipid extraction on biocoal quality. The lipidic content of grinded silkworm pupae was extracted using distilled hexane (95%, ALLCHEM), according to ref 27. The residues underwent a grinding and sieving pretreatment for homogenization (60 mesh) for both feedstocks. The samples were dried at 105 ± 2 °C by using an SL-100/1080I drying oven (Solab, Brazil) and sealed in plastic bags for storage.

### 2.2 Chemical Characterization

Table 2 shows the proximate and ultimate data of the feedstocks. Moreover, the bromatological characterization of the silkworm pupae (Table S1 in the Supporting Information (SM)) was conducted (ABNT NBR 14725–1) for food-grade compositions. The ultimate analysis was performed using the CHN Elemental Analyzer (2400 Series II, PerkinElmer, USA) on raw and torrefied biomass to determine the content of C, H, and N. Oxygen content was determined by difference, i.e., O = 100 −C–H −N– ash.

**Table 2:** Physical Aspect, Proximate, Ultimate, and Calorific Analysis for Raw Silkworm Pupae, Integral Silkworm Pupae (ISP), and the Silkworm Pupae Post Lipidic Extraction, Extracted Silkworm Pupae (ESP)

feedstock	ISP	ESP
moisture	1.17 ± 0.3	1.86 ± 0.2
proximate analysis (%)^a^		
Ash	11.23 ± 0.6	13.20 ± 0.5
fixed carbon	10.68 ± 0.3	14.45 ± 0.3
volatile matter	78.09 ± 1.0	72.35 ± 0.8
fuel ratio	0.14 ± 0.1	0.20 ± 0.1
ultimate analysis (%)^a^		
C	51.36 ± 0.02	40.96 ± 0.03
H	7.91 ± 0.01	6.41 ± 0.04
N	7.82 ± 0.01	11.65 ± 0.01
O^b^	21.69 ± 0.02	27.79 ± 0.02
formula	CH_1.83_O_0.32_N_0.13_	CH_1.86_O_0.51_N_0.24_
calorific analysis (MJ·kg^–1^)^c^		
HHV	23.18 ± 0.04	17.42 ± 0.03

a Dry basis.

b By difference.

c Calculated by eq 2.

A thermogravimetric analyzer (Shimadzu, DTG-60, Japan) determined the ash content (%). The volatile matter (VM, in %) and fixed carbon (FC, in %) were determined following the ABNT NBR 8112/86 standard. The experimental results were uniform between runs, and the relative error was controlled below 3%. Moreover, the fuel ratio (FR) was determined considering the ratio between FC and VM (eq 1).

1 $$\mathrm{FR}=\frac{\mathrm{FC}}{\mathrm{VM}}$$

The Channiwala and Parikh equation^28^ (eq 2) was used to estimate the higher heating value (HHV, in MJ·kg^–1^) of the torrefied biomass waste, as it is widely applied to various biomass types, including lignocellulosic waste and chitinous material (shrimp waste), showing reasonable accuracy.^29^

2 HHV=0.3491+0.3137C+1.1783H+0.1005S−0.1034O−0.0151N−0.0211ASH

### 2.3 Experimental Torrefaction

The ISP and ESP (samples of 3 ± 0.10 g) were treated in triplicate in a torrefaction system (Figure 1b) comprising an alumina recrystallized horizontal tube (inner diameter of 4 cm) placed inside an electrically heated furnace (4 kW). A constant N_2_ (99.999%, White Martins) flow of 1 L·min^–1^ controlled the inert atmosphere. An S-type thermocouple continually controlled the torrefaction temperature. The torrefaction experiments were initially conducted for ISP and ESP, covering the full torrefaction range (180–320 °C) and durations (20–60 min) to characterize both light and severe torrefaction conditions while maintaining a fixed heating rate of 5 °C·min^–1^, a standard parameter in torrefaction studies.^30^

Subsequently, considering the better results of ESP (see Section 3.2) and potential applications of extracted silkworm lipids, including biodiesel production,^31^ eco-friendly grease,^7^ and surfactant^32^ production, ESP was selected for process optimization using RSM (Section 2.4).

The solid yield (SY, in %) was calculated by eq 3, considering the ratio between the mass of torrefied biomass (*m*_biocoal_(*t*)_*T*_, in g) and the initial mass (*m*_initial_, in g) of the sample on a dry basis, according to treatment temperature *T* (°C) and time *t* (min).

3 $$\mathrm{SY}=\frac{m_{\mathrm{biocoal}}{\left(t\right)}_{T}}{m_{\mathrm{initial}}}\times 100$$

The energy densification ratio (ED, dimensionless), energy yield (EY, in %), and energy-mass coefficient index (EMCI, dimensionless) were calculated by eqs 4–6.

4 $$\mathrm{ED}=\frac{{\mathrm{HHV}}_{\mathrm{biocoal}}}{{\mathrm{HHV}}_{\mathrm{raw}}}$$

5 EY=SY×ED

6 EMCI=EY−SY

In this context, EY was calculated exclusively on the basis of the solid torrefied fraction, considering the retained mass and energy densification. Gaseous and vapor-phase losses, as well as thermal input to the system, were not included in the boundary of this calculation. This approach is consistent with standard torrefaction study methodologies and reflects the solid biofuel’s performance rather than the overall process efficiency.

### 2.4 Optimization

The torrefaction temperature and holding time provide changeability in its torrefied products and are allied to the biomass feedstock variability (lignocellulosic and nonlignocellulosic), which requires a comprehensive assessment. Hence, this investigation encompasses an RSM to optimize the torrefaction conditions specifically for the not-yet-explored silkworm feedstock (ESP).

The operating conditions were determined using a design of experiments approach based on a central composite design (CCD) with α = 1.41421. Specifically, a central composite face-centered (CCF) design was employed, given its effectiveness and its reliability in optimizing various processes, including torrefaction.^33,34^

Previous works have conducted RSM to evaluate and optimize lignocellulosic biomass, assessing torrefaction temperature (200–300 °C) and time (20–60 min).^16,34,35^ To the best of our knowledge, no work has applied RSM to silkworm pupae residues. Therefore, a design of experiments (DOE) was developed to explore the full torrefaction range and extend its boundaries given that this is the first investigation of this feedstock in this thermochemical process. The DOE included 13 experiments and assessed the influence of torrefaction temperature (155–325 °C) and treatment time (12–70 min) on ESP, considering the 5 °C·min^–1^ heating rate. Based on previous studies,^30,36^ the present optimization explored SY and HHV. The experimental values were incorporated into the software (Stat-Ease Design-Expert, version 23.1.4) for evaluating the RSM–CCD.

The data collected from the experiments were utilized to obtain a reduced cubic model for SY (eq 7) and a two-factor interaction model for HHV (eq 8) with the highest polynomial degree (without aliasing) to predict and optimize the selected parameters.

7 $$R_{1}=\beta_{0}+\beta_{1}A+\beta_{2}B+\beta_{12}\mathrm{AB}+\beta_{11}A^{2}+\beta_{22}B^{2}+\beta_{122}AB^{2}+\varepsilon$$

8 $$R_{2}=\beta_{0}+\beta_{1}A+\beta_{2}B+\beta_{12}\mathrm{AB}+\varepsilon$$

Here, β_*i*_ represents the constant regression coefficient, *A* and *B* represent input temperature and time (in °C and min) parameters, and *ε* represents the normally distributed residuals, estimated via ANOVA with constant variance assumption, as recommended by RSM guidelines.^37^ The regression models (eqs 7 and 8) were built using stepwise selection in Design-Expert (v23.1.4), retaining only statistically significant terms to avoid overfitting and multicollinearity.^3^

An ANOVA was performed to evaluate the regression models’ accuracy with a confidence level of 95%. Additionally, the three-dimensional (3D) surfaces of each response model were examined to understand how the independent variables and their interactions influence the torrefaction outcomes (*R*_1_–*R*_2_).

The RSM framework allowed for optimization of the torrefaction process to obtain torrefied products as biofuels. Therefore, a statistical-desirability-based optimization was conducted to assess interactions and achieve an optimal outcome when multiple criteria were considered concurrently.

Each response (*Y*_*i*_) can be transformed into a dimensionless value within a range of 0–1, where 1 represents the desired value for a specific response. This value is also known as individual desirability (*d*) and is contingent on whether the response will be maximized or minimized. In this study, as maximized and minimized responses are expressed, their desirabilities were computed using eqs 9 and 10.

9 $$d=\{{\left(\frac{Y_{i}-L_{i}}{H_{i}-L_{i}}\right)}^{s}\begin{array}{l} \mathrm{if}\;Y_{i}<L_{i}\\ if\;L_{i}\leq Y_{i}\leq H_{i}\\ \mathrm{if}\;Y_{i}>H_{i} \end{array}$$

10 $$d=\{{\left(\frac{H_{i}-Y_{i}}{H_{i}-L_{i}}\right)}^{t}\begin{array}{l} \mathrm{if}\;Y_{i}<L_{i}\\ \mathrm{if}\;L_{i}\leq Y_{i}\leq H_{i}\\ \mathrm{if}\;Y_{i}>H_{i} \end{array}$$

where the exponents *s* and *t* are parameters expressing the importance of (*Y*_*i*_) so that the individual desirability aligns closer to the maximum and minimum, respectively, and *L*_*i*_ and *H*_*i*_ represent the minimum and maximum acceptable values for each response, which can be adjusted based on process requirements.^38^ The individual desirability exponents (*s* and *t*) were set to 1, following standard practice to apply a linear desirability function without bias toward the lower or upper limits.^39,40^ This approach ensures a proportional weighting across the optimization range.

Individual desirability can be aggregated into the overall desirability (*D*), as indicated in eq 11. The parameter combination that yields the highest *D* represents the optimal parameters.^38^ The predicted optimal conditions were then replicated to validate the predictive capacity of the overall desirability approach.

11 $$D={{(d}_{1}\times d_{2}\times \cdot \cdot \cdot \times d_{n})}^{1/n}$$

Only the independent variables SY and HHV were considered through optimization to avoid multiple contributions from the calculated parameters. For this purpose, the optimization was delineated to outline the biocoal quality (maximizing SY and HHV) without factoring in system prerequisites, which was maintained in range.

## 3 Results and Discussion

### 3.1 ISP and ESP Torrefaction

#### 3.1.1 Solid Yield

Recent studies have shown that integral silkworm pupal residues contain ∼30% chitin content, highlighting their importance for biomass conversion.^41^ These compositional characteristics directly influence SY trends, particularly considering lipid-extracted silkworm pupae (ESP), which, due to lipid removal, retain a higher % of chitin content.

The thermal degradation of chitin follows a multistep process, beginning with dehydration and structural weakening at 100–250 °C, where water evaporation and the breaking of weak hydrogen bonds occur.^42–44^ As the temperature increases to 250–350 °C, deacetylation of *N*-acetyl-d-glucosamine units releases acetic acid (CH_3_COOH), contributing to a significant mass loss (ca. 30–40%).^42–44^ During this phase, polymer backbone scission generates oligosaccharides and anhydro-sugars, with partial conversion to chitosan-like intermediates.^42–44^

Figure 2 presents the SY of torrefied ISP and ESP. The progressive degradation pattern aligns with the SY trends observed during torrefaction, where the SY decreases proportionally with increasing temperature due to the release of volatile compounds. The SY varied between 95.27 and 78.09% for ISP runs and between 91.84 and 72.06% for ESP runs, with higher SY in ISP due to its lipid content, which may act as a thermal buffer, delaying chitin degradation. Furthermore, ESP exhibited stronger time dependence at 300 °C, which correlates with prolonged volatilization of deacetylation byproducts (Figure 2).

**Figure 2:**
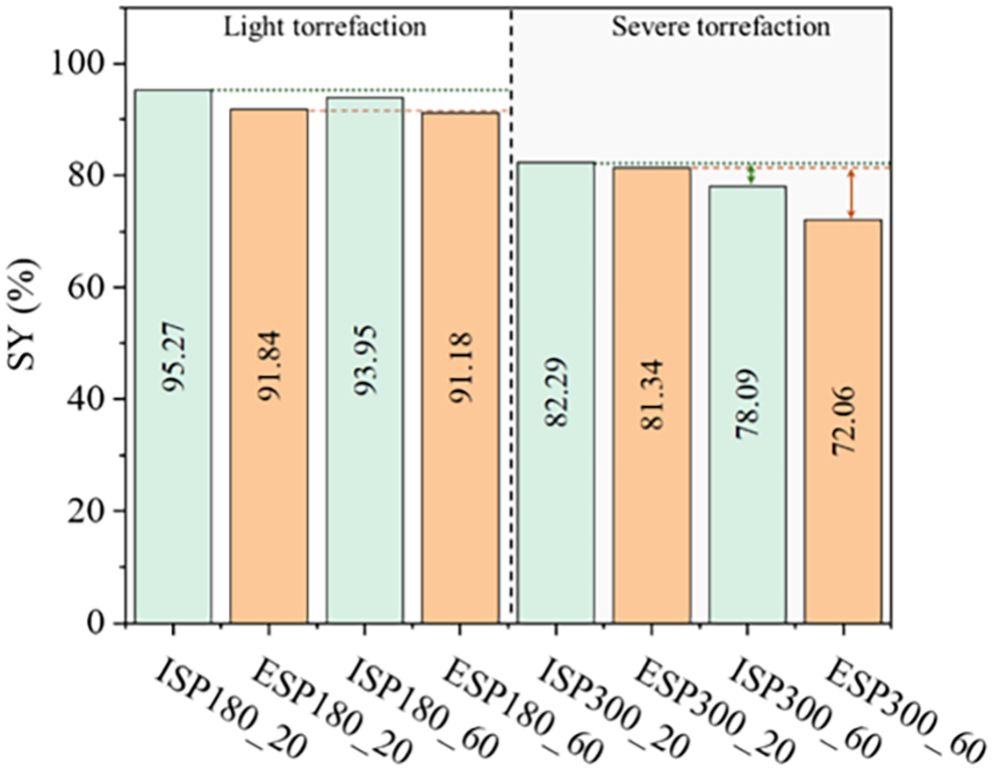
Solid yield (in %) for torrefied integral silkworm pupae (ISP) and extracted silkworm pupae (ESP) at 180 °C (20 and 60 min) and 300 °C (20 and 60 min).

Under severe torrefaction conditions, the SY exhibited a strong time dependence with mass loss increasing significantly with extended residence time. For ISP, the SY decreased from 82.29 to 78.09%, representing a 5.1% increase in mass loss. This effect was even more pronounced in ESP, with SY declining from 81.34 to 72.06%, corresponding to an 11.4% increase in mass loss. This increase indicates that ESP is more susceptible to prolonged thermal exposure than ISP, likely due to its lower lipid content, which accelerates the volatilization of structural components, such as chitin. These results confirm that temperature is the dominant factor in degradation, but longer torrefaction times exacerbate mass loss, particularly for ESP.

As noted in Table 1, no literature has explored pyrolysis or torrefaction treatment for silkworm pupae residues. Therefore, for comparison purposes, literature data from both lignocellulosic and nonlignocellulosic biochars produced through different thermochemical processes (torrefaction, pyrolysis, and hydrothermal carbonization) were considered. The present results showed higher SY compared to torrefied (300 °C) lignocellulosic biomass, which reported values of 44% for rice straw (softwood) and 46% for pinewood (hardwood).^45^

Chitin-rich and lipidic-containing biomasses are often studied as pyrolytic biochar (preparation >300 °C) (see Table 1). Regarding biochar from insect, Różyło et al. reported a 22.9 and 22.3% SY for waste cricket (*Acheta domesticus*) pyrolysis at 500 and 700 °C.^23^ Kannan et al.^46^ investigated the hydrothermal carbonization of shrimp waste and highlighted the impact of temperature and time on hydrochar properties. The hydrochar yield was maximized at a holding temperature of 186 °C and 120 min, achieving a yield of approximately 29%.

Concerning lipidic-containing biomasses, an SY of 63% was achieved for pig manure pyrolyzed at 300 °C, as reported in ref 45. Moreover, pyrolyzed sewage sludge at 300 °C showed a 66% SY.^47^ These comparisons underscore the superior solid retention exhibited by silkworm pupa residues under torrefaction (200 to 300 °C), as demonstrated by the experimental results.

#### 3.1.2 Proximate Properties

Figure 3a shows the ternary diagram of the proximate properties (Table S2), revealing, for the first time, two distinct regions that characterize the torrefied product derived from silkworm pupae. These regions differentiate it from other biomass types, including lignocellulosic materials (spent coffee grounds,^48^ rice straw, eucalyptus, and pinewood^49^), and chitinous feedstocks (shrimp^46^). This distinction highlights the unique compositional attributes of torrefied silkworm pupae compared with conventional biomass sources. The higher VM of ISP might be related to the overall volatility of lipidic extractives, which were removed from ESP samples, which is in line with the literature data.^51^ Higher temperatures promoted VM release, showing higher temperature than time dependence, noticeable for severe torrefaction (300 °C).

**Figure 3:**
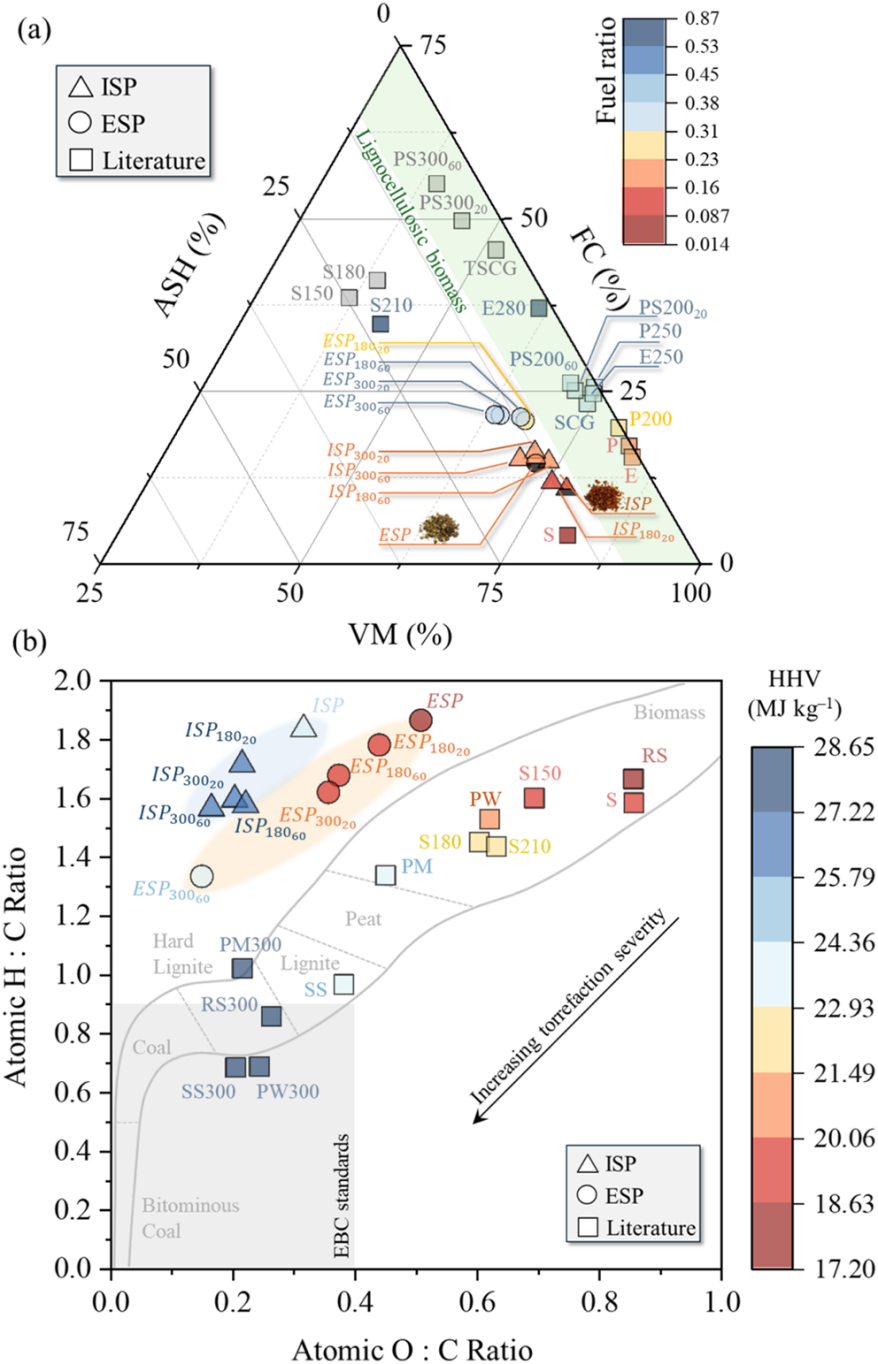
(a) Ternary diagram for ISP and ESP. Raw and torrefied spent coffee ground (SCG and TSCG),^48^ *Pinus elliottii* (P), shrimp residues (S),^46^ and *Eucalyptus sp.* (E) from ref 49. (b) Van Krevelen diagram for the ISP and ESP. Literature comparison: Rice Straw (RS), Pig Manure (PM), Pinewood (PW), and Sewage Sludge (SS) from ref 45. The gray region determines the recommended limits by the European Biochar Certificate (EBC).^50^

FC ranged between 20.61 and 21.46% for ESP and between 11.66 and 16.02% for ISP. The higher FC of ESP might be attributed to the torrefied chitinous chains and the absence of lipidic extracts, which do not provide the organized carbon chains for the FC determination.^52^ The FC increased at higher temperatures (300 °C). The FC response indicates that torrefied ESP is a superior biofuel to torrefied ISP.

The ash content was comparable between both biomasses, ranging from 11.47 to 14.84% for ESP and from 11.48 to 14.91% for ISP. The torrefied ESP and ISP exhibited characteristics like other torrefied biomasses (Figure 3a), particularly in terms of VM and FC at mild torrefaction conditions (∼235 °C). However, the higher ash content distinctly differentiates these biomasses from others.

The correlation between the SY and proximate properties highlights the influence of compositional changes during torrefaction. As temperature and residence time increase, SY decreases due to the release of VM, which is more pronounced in ESP. FC increases with the treatment severity, indicating enhanced carbonization. The stronger SY decline in ESP aligns with its lower VM retention and ash accumulation, emphasizing the role of composition in the mass loss dynamics.

#### 3.1.3 Ultimate Properties

The Van Krevelen diagram, illustrated in Figure 3b, provides a comprehensive analysis of torrefied outcomes and enables a comparison with literature data. Additionally, the ultimate analysis results (C, H, N, and O) are presented in Table S2 of the SM. As expected, the atomic H/C and O/C ratios of torrefied ISP and ESP exhibit a decreasing trend with increasing torrefaction severity, indicating a stronger temperature dependence than the residence time. This trend aligns with the observed variations in SY (Figure 2) and VM (Figure 3a), reinforcing the progressive carbonization process during torrefaction.

The light torrefaction treatments of ESP (180 °C and 20 min) showed a higher proximity to the feedstock. In comparison, severe torrefaction drives the composition toward the left down corner, as observed for ESP treated at 300 °C for 60 min. This condition resulted in a markedly higher degree of deoxygenation and dehydration, yielding elemental compositions comparable to torrefied pig manure at 300 °C (PM300) and approaching those typical of lignite.^45^ This displacement indicates a distinct compositional region compared to ESP and exhibits low similarity to rice straw (RS), pig manure (PM), pinewood (PW), and sewage sludge (SS) from refs 45,53 or established char ranges.^54^ The observed misalignment of ISP within these char ranges can be attributed to its lipid composition, primarily consisting of linoleic and oleic acids (exceeding 85%).^7^ During torrefaction, the oligomerization of fatty acid chains might alter the material’s structural properties, reducing its resemblance to conventional char.

The O/C ratios of torrefied ESP (300 °C, 60 min) presented similar values (∼0.25) to other torrefied lignocellulosic biomasses (rice straw and pinewood).^45^ Meanwhile, a noticeable difference was observed in the H/C ratio, which varied randomly between 1.56 and 1.71 for ISP while steadily declining from 1.78 to 1.33 for ESP. Under severe torrefaction, the torrefied material exhibited higher H/C values than lignocellulosic biomass, such as pinewood (H/C = 0.7 ^45^), highlighting the unique compositional characteristics of silkworm residues and their thermal decomposition.

Torrefaction promoted a higher degree of deoxygenation, presenting an O/C as low as 0.17 and 0.15 for ISP and ESP for severe (300 °C, 60 min). The produced biocoal did not meet the EBC standards (the gray region in Figure 3b), which specify that the molar H/C and O/C ratios must be below 0.7 and 0.4, respectively, to be considered biochar. The distinct clustering of ISP and ESP highlights the influence of the lipid content and thermal severity on elemental composition. ISP’s behavior reflects the stabilizing effect of lipids, while ESP’s trends emphasize enhanced aromaticity and reduced polarity, properties favorable for solid fuel applications. Importantly, the movement of ESP toward the lignite boundary demonstrates its potential as a coal-like biofuel, particularly when torrefied at higher severities.

#### 3.1.4 Energetic Properties

Figure 4a exhibits the HHV for ISP and ESP. Considering the explored torrefaction conditions, the HHV varied between 24.59 and 25.39 and between 18.91 and 23.72 MJ·kg^–1^ for ISP and ESP, respectively. The HHV was enhanced with treatment temperature in an expected energetic densification process, while residence time slightly enhanced this property. This behavior corroborates the previously discussed SY reduction due to the volatile release and carbon fixation (resulting in a lower H/C and O/C).

**Figure 4:**
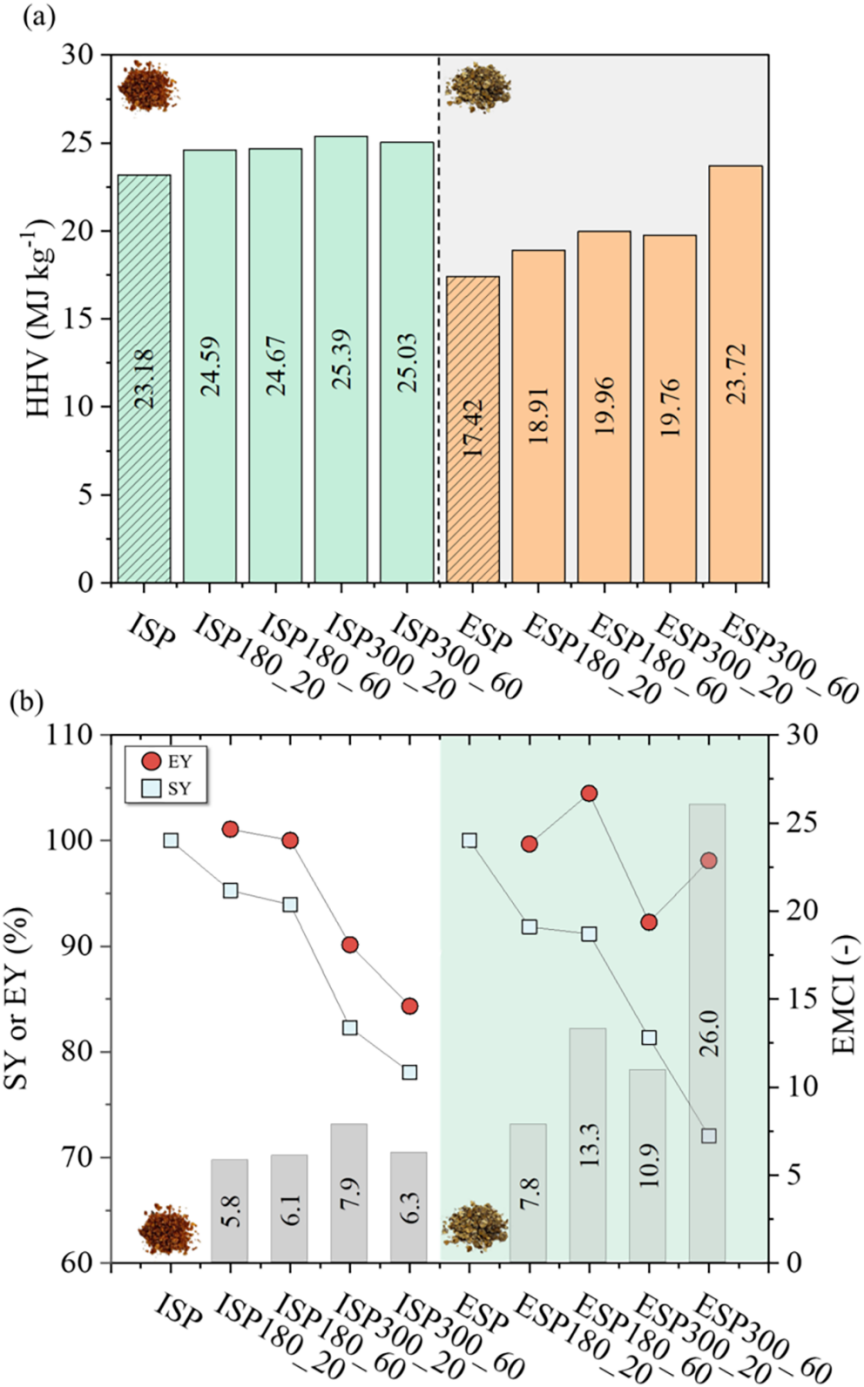
(a) Higher heating value (HHV) for raw and torrefied ISP and ESP. (b) Solid yield (SY), energy yield (EY), and energy-mass coefficient index (EMCI) for torrefied ISP and ESP.

Compared to other biomasses and thermochemical processes, a previous study investigated chicken manure hydrochar, reporting an HHV of 23.9 MJ·kg^–1^ (300 °C, 180 min).^55^ In addition, Krutof et al.^56^ studied the copyrolysis of softwood and mussel shells, obtaining HHV of 28.7 MJ·kg^–1^ for the pure softwood biochar (400 °C) with a reduction of 70–80% of HHV (5.9–9.0 MJ·kg^–1^) for biochar samples with 50 wt % of mussel shells. Moreover, Peng et al.^57^ studied food waste pyrolysis (700 °C), reporting a biochar with HHV of 13.51 MJ·kg^–1^. Furthermore, literature comparison between present results and lignocellulosic biochar (crop waste 17.4–23.9 MJ·kg^–1^ and *Eucalyptus* and pine needle 23–28 MJ·kg^–1^) reveals similar values, therefore showing the potential of its biocoal as solid fuel.^58^

The energy densification ratio (ED), or HHV enhancement factor, is a severity index related to HHV before and after the thermal treatment, where an ED > 1 represents an energetic densification post-torrefaction.^59^ ED values for ESP and ISP are shown in Figure 4b. ED was lower for ISP (1.06 and 1.10 in Table S2) compared to ESP runs, which ranged between 1.09 and 1.36, highlighting an upgrade of up to 36.14% for ESP obtained with severe torrefaction (300 °C and 60 min).

Kung and co-workers studied the torrefaction of pine shavings, hay, and rice husk at 300 °C for 30 min, resulting in ED values of 1.16, 1.21, and 1.10, respectively.^59^ Silveira et al. evaluated the *Eucalyptus grandis* torrefaction at 210–290 °C for 10–70 min, obtaining ED values between 1.002 and 1.101.^60^ These findings highlight the effectiveness of torrefaction in enhancing energy densification, particularly for ESP, reinforcing its potential for bioenergy applications.

The EY represents the energy content retained in the torrefied solid product. High values of EY indicate low mass losses or a high ED through torrefaction (eq 5). Higher EY values indicate either minimal mass loss or significant energy densification during torrefaction. For the ISP, the highest EY (101.08%) was observed at 180 °C for 20 min, while the lowest EY (84.34%) occurred at 300 °C for 60 min (see Figure 4b). For ESP, the highest EY (104.48%) was recorded at 180 °C for 60 min, whereas the lowest (92.27%) was found at 300 °C for 20 min. Despite ESP exhibiting a higher ED, ISP maintained a higher EY due to its superior SY (Figure 2), suggesting that the lipid content in ISP helps preserve mass during thermal treatment.

A decline in EY was observed as torrefaction severity increased, particularly for ISP at elevated temperatures and extended residence times. However, under mild torrefaction conditions (<300 °C), exceptionally high EY values—exceeding 100%—were achieved, particularly for ESP, with a maximum of 104.48% at 180 °C for 60 min. EY is calculated by multiplying the solid mass yield (SY) by the HHV ratio between torrefied and raw biomass, thus reflecting energy densification within the remaining solid fraction. Similar outcomes have been widely reported in the literature. Silveira et al.^61^ recorded EY values ranging from 64.57 to 100.07% for deoiled pequi seed waste. Rago et al.^62^ achieved 113.2% EY from textile residues at 225 °C and 180 min, while Chin et al.^63^ observed values above 130% for oil palm biomass torrefied between 250 and 280 °C. Martins et al.^64^ reported EY of 111% for *Cistus ladanifer* torrefied at 350 °C for 30 min. More recently, Milovanov et al.^65^ demonstrated EY of 102.1% for sunflower husk torrefied at 220 °C in a fluidized bed of superheated water vapor, with HHV increasing from 19.0 to 20.9 MJ·kg^–1^ and mass yield exceeding 90%.

Although seemingly counterintuitive, these results are thermodynamically consistent, as EY considers only retained energy in the solid and does not account for energy carried by volatiles or input losses. Hence, energy yields greater than 100% reflect the selective volatilization of low-energy compounds and the enrichment of carbon-dense structures within the remaining solid, enhancing its utility as a biofuel.

The energy-mass coefficient index (EMCI) represents the difference between EY and SY, indicating a desired condition in the torrefaction products. The values of EMCI for ESP (7.85–26.04) runs were higher than ISP (5.81–7.85) runs due to effect propagation given overall energy densification on ESP samples, corroborating ESP as better biochar for biofuel applications (Figure 4b). Among all treatments, the highest EMCI value (26.04) was obtained for ESP torrefied at 300 °C for 60 min, demonstrating the most effective energy densification under prolonged thermal exposure. Similarly, the highest EMCI value (7.85) for ISP was recorded at 300 °C for 20 min, indicating that although energy densification occurred, its extent was significantly lower than that of ESP.

Compared to the torrefaction of lignocellulosic material literature, Silveira et al.^16^ reported EMCI values ranging from 2 to 6 for the torrefaction of urban forest waste, conducted at temperatures of 225–275 °C for 60 min. Lu et al.^66^ conducted torrefaction on oil palm fiber (OPF) and *Eucalyptus* in different gaseous environments—nitrogen and air. Their findings indicated that when torrefying OPF, the highest EMCI values (optimal torrefaction conditions) were 19.0 in nitrogen and 7.7 in air, achieved at 300 and 250 °C, respectively.^66^ Similarly, *Eucalyptus* torrefaction showed optimal conditions at 325 °C in nitrogen (with an EMCI value of 18.9) and at 275 °C in air (with an EMCI value of 14.0),^66^ aligning with the current study.

Evaristo et al.^67^ investigated the pyrolysis (500–700 °C, for 60 min) of spent coffee grounds and brewers’ spent grains, revealing EMCI values ranging from 5 to 10 and from 15 to 23, respectively. Meanwhile, Carvalho et al.^68^ examined hydrothermal carbonization (150–220 °C, for 60 min) of *E. grandis* sawdust, showcasing EMCI values fluctuating between 2.5 and 16.37, like torrefied ESP.

In summary, the superior EMCI values achieved by ESP, particularly under severe torrefaction, underscore its enhanced energy densification and suitability as a biofuel feedstock. The significantly higher EMCI of ESP compared to that of ISP confirms the benefits of lipid extraction in promoting favorable fuel properties. These findings provide a strong foundation for subsequent process optimization, aiming to maximize the energetic and material performance of ESP-derived biocoal.

### 3.2 ESP Torrefaction Optimization

The ESP torrefaction’s experimental responses (SY and HHV) are compiled in Table S3, considering the mean of at least two duplicate runs (error was controlled below 3%). The three-dimensional (3D) and contour plots of SY and HHV (Figure 5) were plotted with the obtained eq 12 (reduced cubic model) and eq 13 (two-factor interaction model). These equations present the prediction equations of SY and HHV considering only the coefficients of statistically significant input variables of ANOVA results (Table 3) in terms of coded factors for temperature in °C (*A*) and time (*B*) (see Table S4).

12 $$\mathrm{SY}=86.61-9.85A-2.29B-2.15\mathrm{AB}-4A^{2}-1.4B^{2}+2.45AB^{2}$$

13 HHV=20.57+1.18A+1.09B+0.7253AB

**Figure 5:**
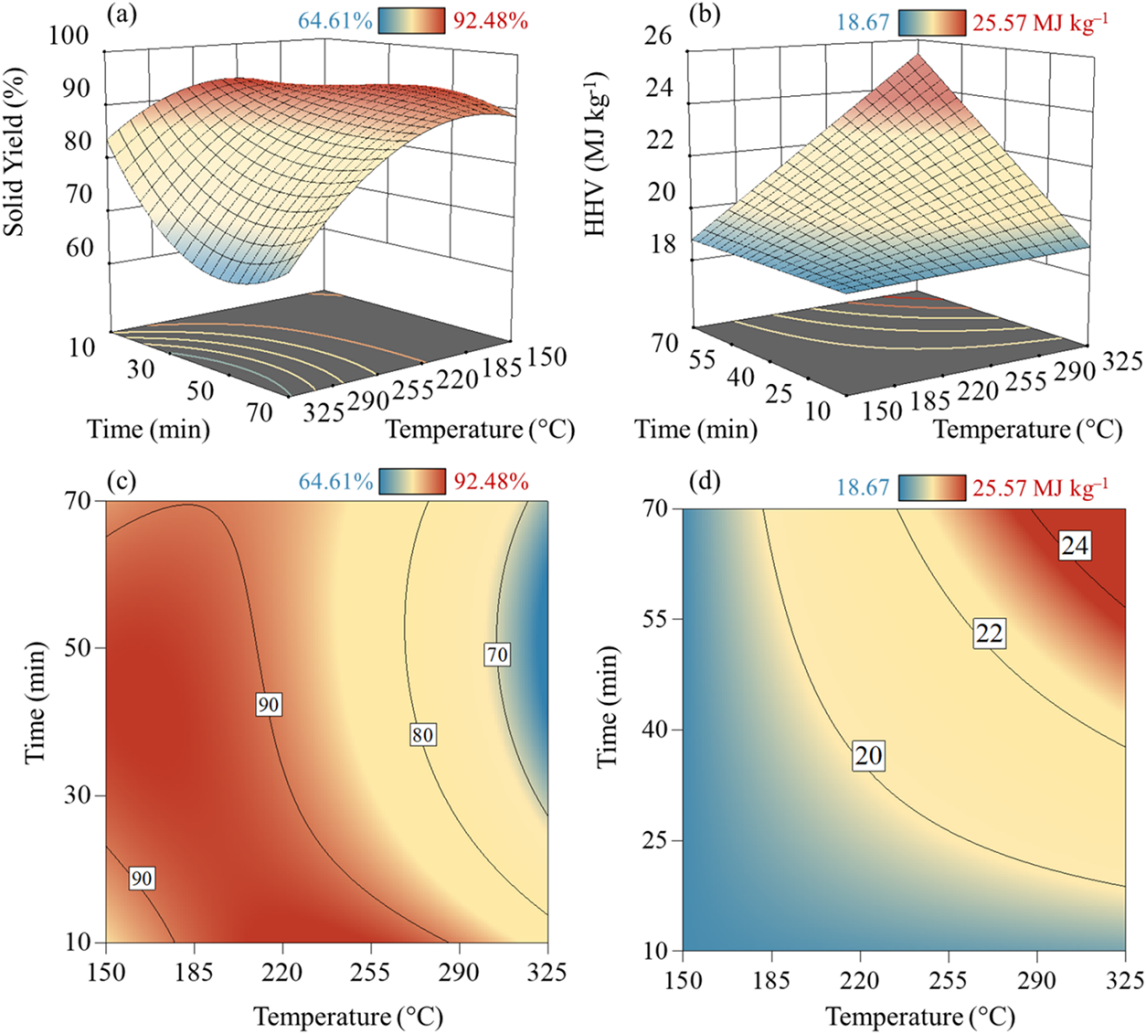
(a) ESP response surface for (a) SY (%) and (b) HHV (MJ·kg^–1^) considering the model’s independent parameters: *A* (temperature in °C) and *B* (time in min).

**Table 3:** ANOVA: Reduced Cubic Model for SY (%) and Two-Factor Interaction (2FI) Model for HHV^a^

source	sum of squares	df^b^	mean of square	*F*-value	*p-*value	
SY						
**model**	795.3	6	132.55	517.2	0.0001	*
*A*. temperature	388.31	1	388.31	1515.12	<0.0001	*
*B*. time	41.82	1	41.82	163.17	0.001	*
*AB*	18.55	1	18.55	72.38	0.0034	*
*A* ^2^	72.99	1	72.99	284.8	0.0005	*
*B* ^2^	9.02	1	9.02	35.18	0.0096	*
*AB* ^2^	12	1	12	46.82	0.0064	*
residual	0.7689	3	0.2563			
lack of fit	0.3631	2	0.1815	0.4474	0.7265	
pure error	0.4058	1	0.4058			
cor total	796.07	9				
***R* ^2^**	0.999	**adjusted *R* ^2^**	0.9971	**predicted *R* ^2^**	0.9918	
**HHV**						
**model**	22.76	3	7.59	71.27	<0.0001	*
*A*. Temperature	11.16	1	11.16	104.86	<0.0001	*
*B*. Time	9.49	1	9.49	89.19	<0.0001	*
*AB*	2.1	1	2.1	19.77	0.0043	*
**residual**	0.6386	6	0.1064			
Lack of Fit	0.6146	5	0.1229	5.14	0.3226	
Pure Error	0.0239	1	0.0239			
**cor total**	23.4	9				
***R*** ^**2**^	0.973	**adjusted *R*** ^**2**^	0.9591	**predicted *R*** ^**2**^	0.9305	

a Statical significance (*p* < 0.05).

b Degree of freedom.

Eqs (S1) and (S2) in SM also present the equations regarding actual values for temperature and time.

#### 3.2.1 Solid Yield

Figure 2a illustrates the response surface plot of the SY for the ESP. Eq 12 depicts negative values for β_1_ (−9.85) and β_2_ (−2.29), thus negatively affecting SY and suggesting a higher temperature dependence than the residence time, as illustrated in Figure 5.

For ESP runs, the experimental SY varied between 72.06 and 92.48% (Table S3). The SY reported an expected tendency, with a decreasing proportion as the torrefaction temperature increases. Meanwhile, the SY time dependence slightly affected light torrefaction, which was more pronounced with severe torrefaction. The positive term of the squared time coefficient (*B* ^2^ with β_22_ of 1.4) suggests that stabilization of SY reduction is attempted.^15^ Figure 5 shows steadying around 40 and 45 min for ESP, with no significant reduction in SY for longer residence times, especially for mild torrefaction (235–275 °C).

#### 3.2.2 Higher Heating Value

Intrinsically related to its ultimate composition, the ESP runs showed HHV (18.67–23.72 MJ·kg^–1^), with maximum HHV attempted at severe temperatures (300 °C, 60 min). Results align with the HHV values of torrefied biomasses (Figure 3b). The HHV was enhanced with treatment temperature (β_1_ of 1.18 in eq 13) in an expected energetic densification process, with a smaller influence of residence time (β_2_ of 1.09 in eq 13). This behavior corroborates the previously discussed SY reduction due to the volatile release and carbon fixation (resulting in a lower H/C and O/C) and an increased HHV.^69,70^

#### 3.2.3 Model Performance

The predicted and actual (experimental) data for the obtained responses are displayed in Figure 6, which shows the regression adjustments (*R* ^2^) for the responses. Both models (quadratic and cubic) demonstrate statistical significance (*p* < 0.005). High regression adjustment values were observed despite sample heterogeneity, with *R* ^2^ > 0.973. Results are in line with the literature that applied RSM to investigate SY of pyrolyzed bamboo biochar (*R* ^2^ of 0.95),^71^ SY from spent coffee ground biochar (*R* ^2^ of 0.87),^72^ and SY and HHV of oxidative torrefaction of microalga (*R* ^2^ of 0.96 and 0.90).^15^

**Figure 6:**
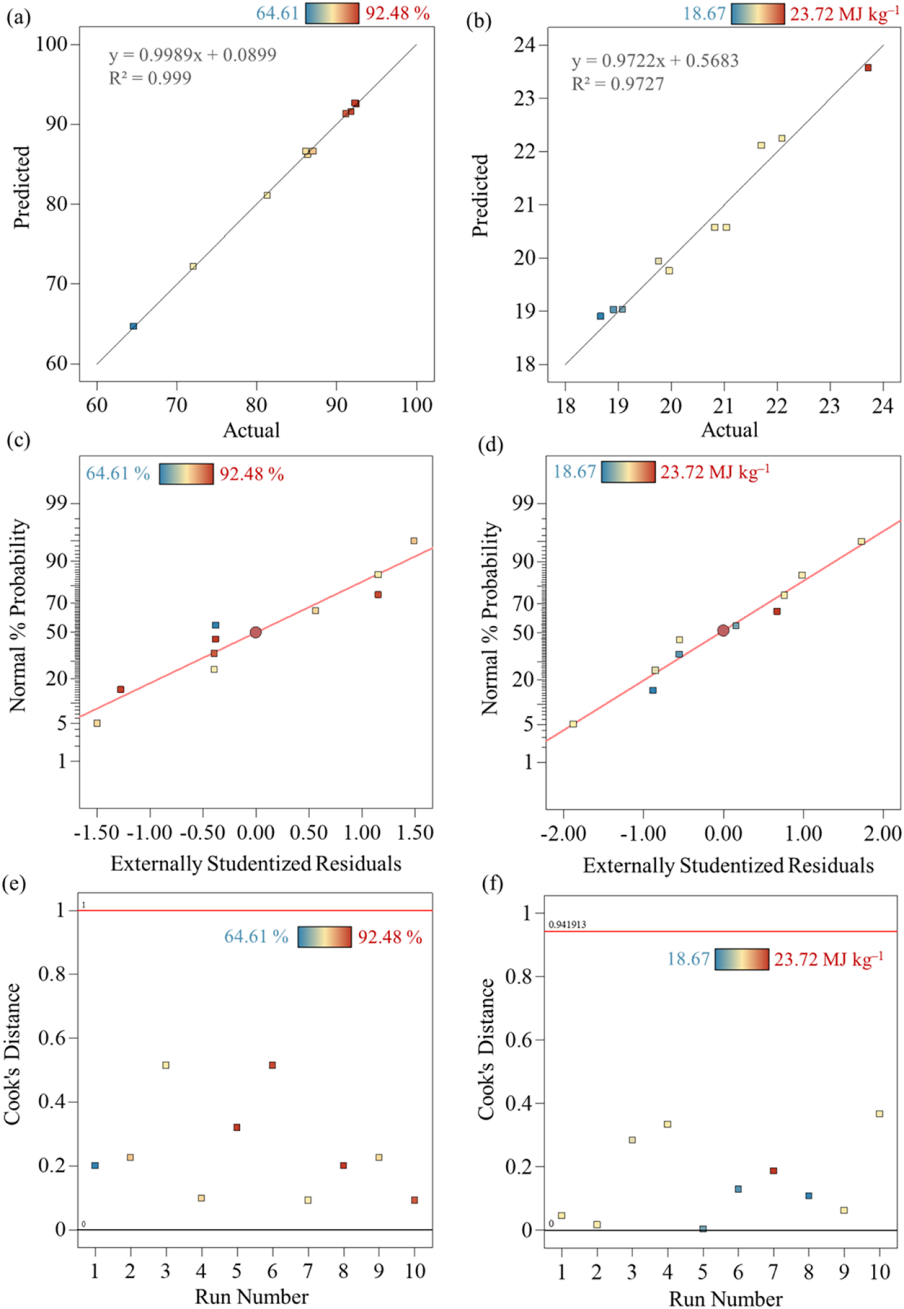
Correlation of predicted and experimental data. (a) SY and (b) HHV. Normal plot of residuals. (c) SY and (d) HHV. Cook’s distance. (e) SY and (f) HHV.

The normal probability plot, obtained using the RSM technique, evaluates the model efficiency by comparing residuals (*X*-axis) to percent values (*Y*-axis). Residuals represent the difference between experimental (exact) and predicted values. Scattered points on the plots indicate model accuracy, with closer alignment to the theoretical normal distribution line, signifying higher model efficiency. Figure 6c,d illustrates these plots, showing the model’s performance in predicting SY and HHV, thus confirming the model’s robustness and reliability.

Figure 6e,f shows the Cook’s distance plots for the various output responses (SY and HHV), indicating that there are no highly influential data points with a Cook’s distance value exceeding 1. This suggests that the regression models for predicting these responses are robust and not overly influenced by any single observation. The low Cook’s distance values across all plots indicate that the models provide stable predictions and that the data points consistently influence the regression outcomes. This enhances the reliability of the models in predicting the various parameters related to the torrefaction process.

#### 3.2.4 Optimal Condition

The optimum solutions are displayed in Table 4. By maximizing SY and HHV, ESP feedstock was selected as the optimum feedstock torrefied at 255 °C for 60 min (desirability of 0.676). The torrefied product indicated an SY of 83.13% with an HHV of 22.12 MJ·kg^–1^. With the obtained values, it is possible to calculate the related ED (eq 4), EY (eq 5), and EMCI (eq 6), which presented 1.27, 104.74%, and 21.61, respectively.

**Table 4:** Optimum Conditions and Predicted Values at Optimal Torrefaction of ESP

parameters		goal
operational conditions		
*A*: temperature	255.00 °C	-
*B*: time	60 min	-
predict properties		
solid yield (%)	83.13	maximize
HHV (MJ·kg^–1^)	22.12	maximize
desirability	0.676	
calculated		
energy yield (%)	104.72	-
ED	1.27	-
EMCI	21.61	-

The optimization of torrefaction conditions using response surface methodology (RSM) has been extensively studied across various lignocellulosic and nonlignocellulosic biomass feedstocks. The diversity of optimal torrefaction conditions reflects the strong dependence on the feedstock composition and process parameters.

Comparing the studies of RSM applied to optimize torrefaction, agricultural residues such as corncob required high-severity torrefaction (300 °C for up to 30 min) to maximize biocoal yield (62.30%) and calorific value (23.37 MJ·kg^–1^),^36^ while urban forest waste (UFW) achieved a high biocoal yield (87.82%) at HHV (22.13 MJ·kg^–1^) at a milder condition of 256 °C for 41 min. Similarly, waste cork powder underwent torrefaction at 275 °C for 60 min, producing a biocoal with 69.5% yield and a higher heating value (HHV) of 23.3 MJ·kg^–1^, comparable to corncob biocoal.^30^ Notably, pequi seeds exhibited one of the highest biocoal yields (82.11%) and an HHV of 24.11 MJ·kg^–1^ under optimized conditions of 274 °C for 42 min, further emphasizing the potential of agro-industrial waste for biofuel applications. Additionally, microalgae-derived biocoal demonstrated exceptional yields exceeding 90% under oxidative and inert conditions at relatively low temperatures (∼201 °C), highlighting the feasibility of torrefaction for algae-based biofuels.^15^ The findings indicate that feedstock-specific optimization is crucial for achieving desirable biocoal properties, with key implications for industrial-scale biofuel production, energy efficiency, and environmental impact reduction.

## 4 Limitations and Prospects

The present assessment encompasses proposing, evaluating, and optimizing silkworm pupae feedstock, considering its integral form and lipid-extracted. The investigated fuel properties allowed new insight into the valorization of these agro-industrial residues as biofuel. Moreover, it provided optimized operational conditions.

The proximate properties of torrefied silkworm pupae indicate a significant modification in the proximate properties that might promote different combustion behaviors. Therefore, future research should address an in-depth investigation, including molecular level chemistry, numerical modeling of combustion behavior (isoconversional and DAEM models), and provide combustion kinetics and thermodynamic parameters of torrefied biomass.^73^

Exploring alternative torrefaction methods, such as oxidative^15,74^ and catalytic torrefaction^75–77^ and different thermochemical routes (hydrotreatment^70,78^ and pyrolysis^20,79^ ) is essential in pursuing cost reduction, simplifying operational processes, and understanding the potential valorization of silkworm pupae residues.

Additional efforts are also required to advance the upscaling of torrefaction systems from laboratory conditions to industrial applications. In particular, future research should include expanded physical and bulk characterization of raw and torrefied powders, such as particle size distribution, morphology, true and bulk densities, and flowability, which are pivotal to improving material handling, feeding, and reactor design.^80–82^ Nonetheless, these analyses alone are not sufficient. Further progress requires the development of predictive numerical models that incorporate both mass and heat transfer limitations, supporting the extrapolation of laboratory results to industrial reactors. In addition, experimental validations in pilot- or real-scale torrefaction systems are essential to strengthen model accuracy and capture operational complexities.

Moreover, incorporating torrefaction as a pretreatment step within the valuation pathway may introduce additional costs. In addition, considering alternative valorization routes for different biocoal applications could lead to enhanced aggregated value and improved environmental performance.^83^ Therefore, future research should prioritize cost assessments using exergoeconomic and advanced sustainability evaluations through life cycle assessment^84^ and exergoenvironmental analyses.^85^

## 5 Conclusions

This study explored the torrefaction of sericulture agro-industrial waste, demonstrating its potential as a sustainable waste-to-energy strategy. By comparing integral silkworm pupae (ISP) and lipid-extracted silkworm pupae (ESP), the results highlighted that ESP exhibits superior biocoal properties, with a higher fixed carbon content, enhanced energy densification, and improved fuel characteristics. The removal of lipids before torrefaction not only enhances biocoal quality but also allows for the recovery of valuable oils, which can be further utilized in biorefinery applications such as biodiesel production, eco-friendly lubricants, or surfactants. This integrated approach maximizes resource efficiency by valorizing the solid residue as biocoal and extracted lipids for alternative industrial applications.

The optimization of ESP torrefaction using RSM identified 255 °C for 60 min as the optimal condition, yielding a high-quality biocoal with a solid yield of 83.13% and a heating value of 22.12 MJ·kg^–1^. These findings confirm that the torrefaction of silkworm pupae can provide a viable and efficient biofuel alternative while reducing agro-industrial waste disposal challenges. From an environmental perspective, this strategy contributes to emissions reduction and circular economy integration. Economically, the dual valorization of torrefied biomass and lipid extracts enhances the profitability of sericulture residues, supporting decentralized bioenergy solutions. Future research should focus on combustion kinetics, large-scale feasibility, and the environmental footprint of this process to reinforce its potential for sustainable bioenergy production.

## Supplementary Material



## Notes

Bromatological data of silkworm pupae and soybean (Table S1); experimental torrefaction results and characterization data for ISP and ESP (Tables S2 and S3); design of experiments (Table S4); and prediction equations for response surface methodology (Equations S1 and S2) (PDF)

## Index Summary

ABNT: Brazilian association of technical standards
ANOVA: analysis of variance
CCD: central composite design
CCF: composite face-centered
DOE: design of experiment
E: *Eucalyptus sp.*
ED: energy densification
EMCI: energy-mass coefficient index
ESP: extracted silkworm pupae
EY: energy yield
FC: fixed carbon
FR: fuel ratio
HHV: higher heating value
HTC: hydrothermal carbonization
ISP: integral silkworm pupae
NBR: Brazilian technical standards
P: *Pinus elliottii*
PM: pig manure
PW: pinewood
RS: rice straw
RSM: response surface methodology
SCG: spent coffee ground
SM: supporting information
SP: silkworm pupae
SS: sewage sludge
SSA: specific surface area
SY: solid yield
VM: volatile matter
