# Supplementary material for: Torrefaction of Sericulture Agro-Industrial Waste for Sustainable Waste-to-Resource Solutions
Source: ACS Omega. 2025 Jun 13;10(25):27223–37. doi: 10.1021/acsomega.5c02508 (PMC12235973; doi:10.1021/acsomega.5c02508)
Supplement: Supplementary file 1 [file ao5c02508_si_001.pdf]

Torrefaction of sericulture agro-industrial waste for sustainable waste-to-resource solutions

Edgar A. Silveira [a*], Romulo C. Dutra [c], Júlia R. Vargas [b,c], Jefferson S. Oliveira [d], Paulo A. Z. Suarez [c], Grace F. Ghesti [b]

a. University of Brasília, Mechanical Sciences Graduate Program, Laboratory of Energy and Environment, Brasilia-DF, 70910-900, Brazil.

b. University of Brasilia, Chemistry Institute, Laboratory of Brewing Bioprocesses and Catalysis to Renewable Energy, Brasilia-DF 70910-900, Brazil.

c. University of Brasilia, Chemistry Institute, Laboratory of Materials and Fuels, Brasilia, DF 70910-900, Brazil.

d. Instituto Federal de Educação, Ciência e Tecnologia de Brasília, Campus Gama, Brasília, DF 72444-240, Brazil

[*]Corresponding author
E-mail: [*]edgar.silveira@unb.br

Supplementary material

The data/information in this document is the supplementary material for the paper Torrefaction of sericulture agro-industrial waste for sustainable waste-to-resource solutions

2. Material and Methods

2.2 Chemical characterization

The silkworm pupae's bromatological characterization (Table S1) was performed following the ABNT NBR 14725-1 for food-grade compositions.

Table S1. Bromatological data for dehydrated silk pupae. Soybean grains for comparison.

**Sample**	**Silkworm pupae**	**Soy beans** [b]
Dry matter (g) [a]	944.46	904.40
Inorganic matter (g) [a]	47.79	58.60
Organic matter (g) [a]	952.21	845.80
Crude protein (g) [a]	520.47	435.60
Lipidic content (g) [a]	285.55	230.69

[a] Material dried at 105 °C for 24 h (g (1000 g)$^{-1}$); [b] Genetically modified soybeans from variety Glycine max (L.) Merril – (CAC-1). Adapted from [1].

3. Results and Discussion

Table S2. Torrefaction results of ISP and ESP

		ISP				ESP			
Properties		180 °C 20 min	180 °C 60 min	300 °C 20 min	300 °C 60 min	180 °C 20 min	180 °C 60 min	300 °C 20 min	300 °C 60 min
SY	(%)	95.27	93.95	82.29	78.09	91.84	91.18	81.34	72.06
**Proximate** [a]									
ASH	(%)	12.56	11.48	12.53	14.91	11.47	11.77	14.18	14.84
VM	(%)	75.77	73.85	71.44	70.07	67.92	67.11	64.44	63.7
FC	(%)	11.66	14.66	16.02	15.03	20.61	21.12	21.38	21.46
**Ultimate** [a]									
C	(%)	55.60	56.24	56.05	56.40	44.23	46.81	46.79	56.15
H	(%)	7.44	7.44	8.06	7.41	6.61	6.59	6.36	6.28
N	(%)	9.29	8.22	7.24	8.83	11.72	11.47	10.39	11.51
O	(%)	15.11	16.62	16.12	12.46	25.97	23.36	22.28	11.22
H/C	-	1.59	1.58	1.71	1.56	1.78	1.68	1.62	1.33
O/C	-	0.20	0.22	0.22	0.17	0.44	0.37	0.36	0.15
**Energetic**									
HHV	$MJ \cdot kg^{-1}$	24.59	24.67	25.39	25.03	18.91	19.96	19.76	23.72
ED	-	1.06	1.06	1.10	1.08	1.09	1.15	1.13	1.36
EY	(%)	101.08	100.02	90.14	84.34	99.68	104.48	92.27	98.11
EMCI	-	5.81	6.07	7.85	6.25	7.85	13.30	10.93	26.04

[a] dry-basis.

Table S3. Experimental design describing independent variables and response results. Torrefaction temperature (A, 180–300 °C), treatment time (B, 20–60 min).

**Treatment**		SY	ASH	VM	FC	C	H	N	O	H/C	O/C	HHV	ED	EY	EMCI
**Temp.**	**Time**	(%)	(%)	(%)	(%)	(%)	(%)	(%)	(%)	-	-	$MJ \cdot kg^{-1}$	-	(%)	-
**156**	**40**	92.48	10.62	69.13	20.25	44.33	6.45	11.64	26.96	1.73	0.46	18.67	1.07	99.10	6.62
**180**	**20**	91.84	11.47	67.92	20.61	44.23	6.61	11.72	25.97	1.78	0.44	18.91	1.09	99.68	7.85
**180**	**60**	91.18	11.77	67.11	21.12	46.81	6.59	11.47	23.36	1.68	0.37	19.96	1.15	104.48	13.30
**240**	**12**	92.30	13.05	68.96	17.99	44.47	6.55	11.93	24.00	1.76	0.41	19.08	1.10	101.09	8.79
**240**	**40**	87.06	11.11	68.38	20.51	46.89	6.27	11.33	24.41	1.59	0.39	20.82	1.12	97.84	10.78
**240**	**40**	86.16	11.65	68.75	20.03	46.77	6.46	11.10	24.03	1.65	0.39	21.04	1.14	97.87	11.71
**240**	**69**	86.39	13.76	67.69	18.55	50.49	6.53	12.81	16.42	1.54	0.24	21.70	1.25	107.62	21.23
**300**	**20**	81.34	14.18	64.44	21.38	46.79	6.36	10.39	22.28	1.62	0.36	19.76	1.13	92.27	10.93
**300**	**60**	72.06	14.84	63.70	21.46	56.15	6.28	11.51	11.22	1.33	0.15	23.72	1.36	98.11	26.04
**325**	**40**	64.61	14.93	58.62	26.45	54.64	5.49	11.63	13.32	1.20	0.18	22.09	1.27	81.93	17.32

Table S4. Design of experiments (DOE) of torrefaction experiments considering a categorical central composite design with α = 1.41421.

Variable	Unit	Coded Factors				
		**-1.414**	**-1**	**0**	**+1**	**+1.414**
Temperature (A)[a]	°C	155.2	180	240	300	324.9
Time (B)[a]	min	11.7	20	40	60	68.3
**Model responses** [b]						
$\boldsymbol{R_1}$ $\boldsymbol{R_2}$						
SY HHV						

[a] Input Variables; [b] Output Variables.

$$SY = 15.863 + 0.604A + 1.995B - 0.010\beta_{12}AB - 0.001\beta_{11}A^2 - 0.021\beta_{22}B^2 + 0.0001AB^2 + \varepsilon \quad \text{(S1)}$$

$$HHV = 19.474 - 0.004A - 0.091B + 0.001AB + \varepsilon \quad \text{(S2)}$$

References

(1) Mendes, F. Q.; de Almeida Oliveira, M. G.; Cardoso, L. R.; Costa, N. M. B.; SantAna, R. de C. O. Digestibilidade Proteica e Caracterização Bromatológica de Linhagens de Soja Com Ausência Ou Presença Do Inibidor de Tripsina Kunitz e Das Isozimas Lipoxigenases. *Bioscience Journal* **2007**.

(2) Bezerra, M. A.; Ferreira, S. L. C.; Novaes, C. G.; Santos, A. M. P. dos; Valasques, G. S.; Cerqueira, U. M. F. da M.; Alves, L. P. dos S. Talanta Simultaneous Optimization of Multiple Responses and Its Application in Analytical Chemistry – A Review. **2019**, *194* (August 2018), 941–959.

(3) Bezerra, M. A.; Ferreira, S. L. C.; Novaes, C. G.; Santos, A. M. P. dos; Valasques, G. S.; Cerqueira, U. M. F. da M.; Alves, L. P. dos S. Talanta Simultaneous Optimization of Multiple Responses and Its Application in Analytical Chemistry – A Review. **2019**, *194* (August 2018), 941–959.
